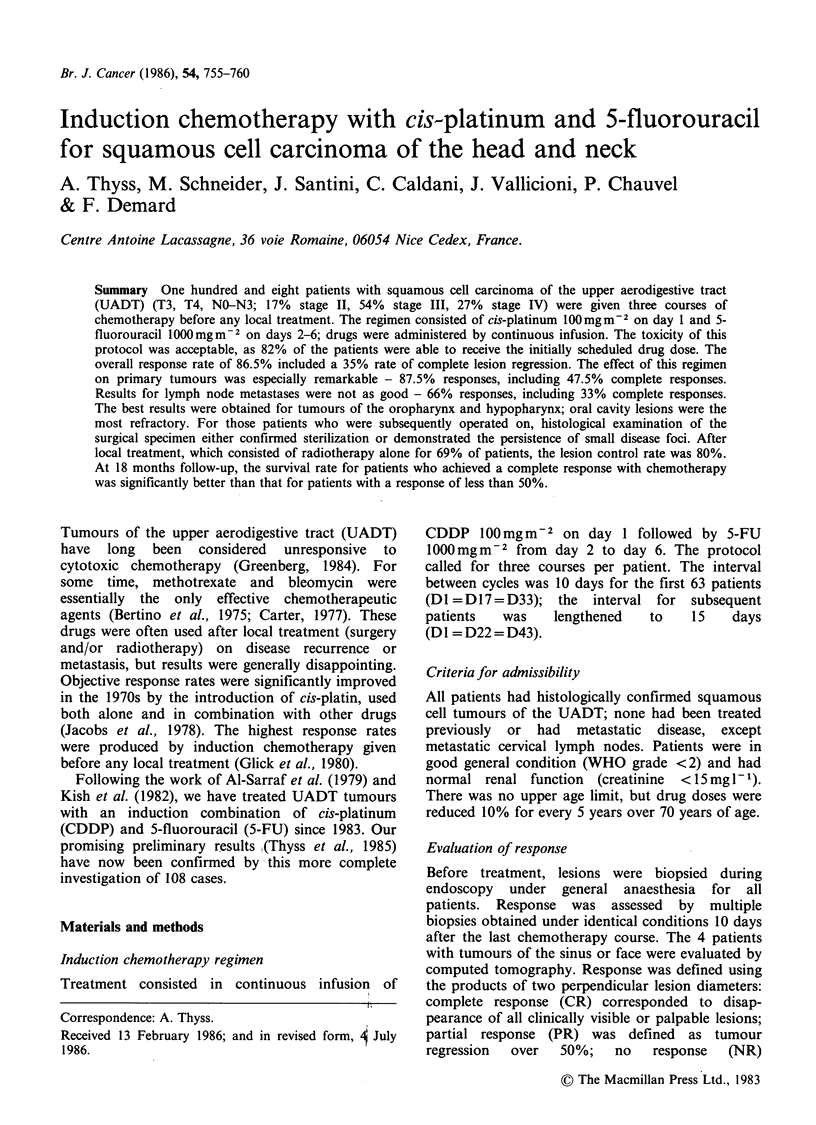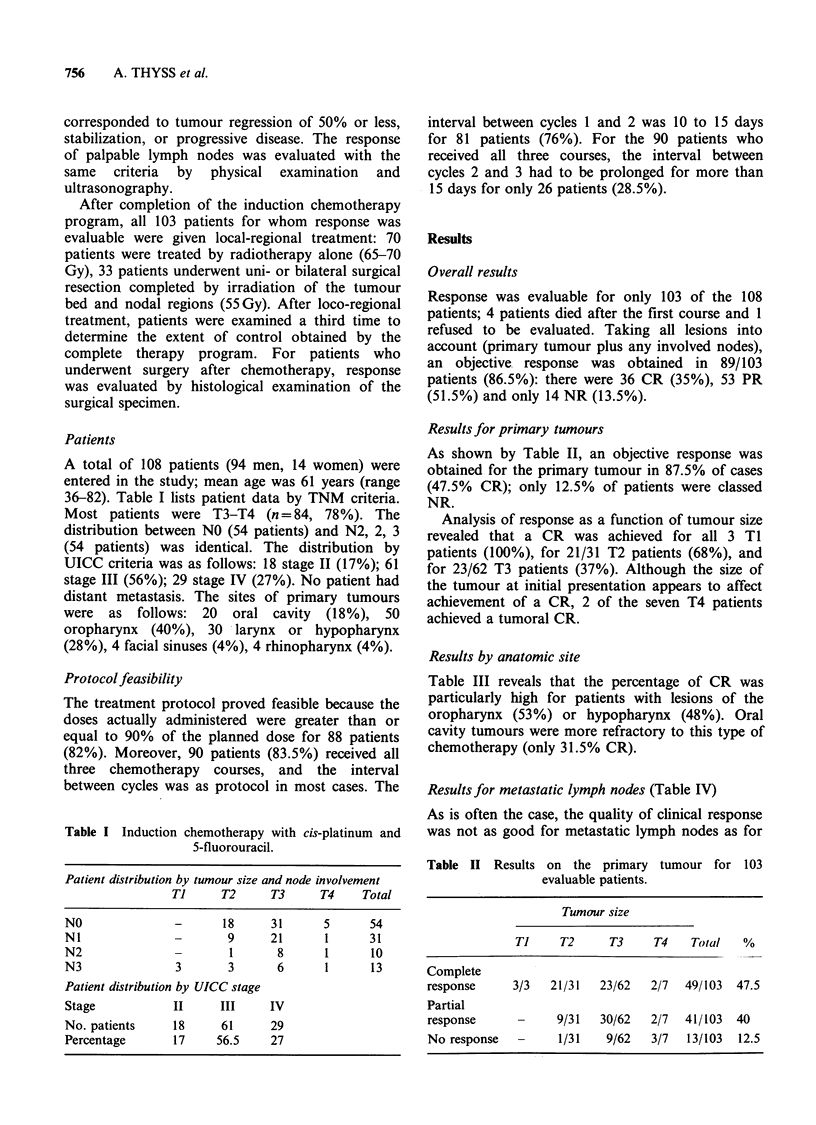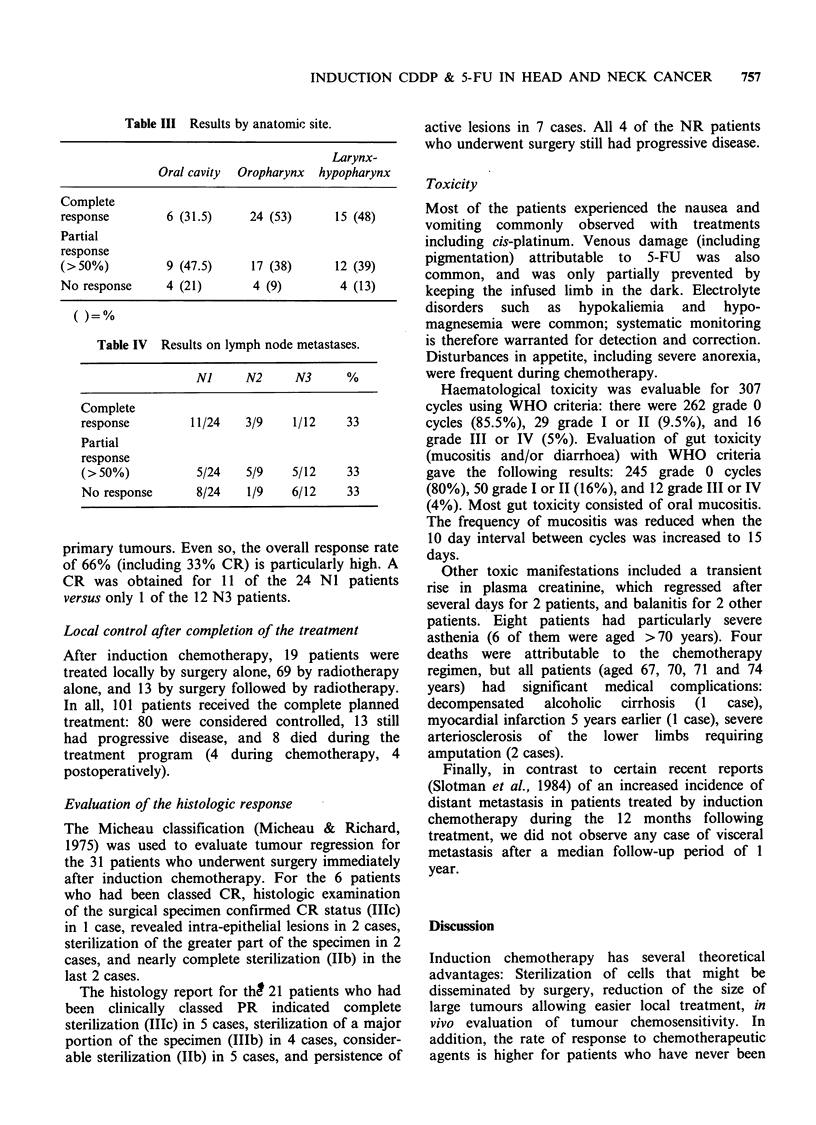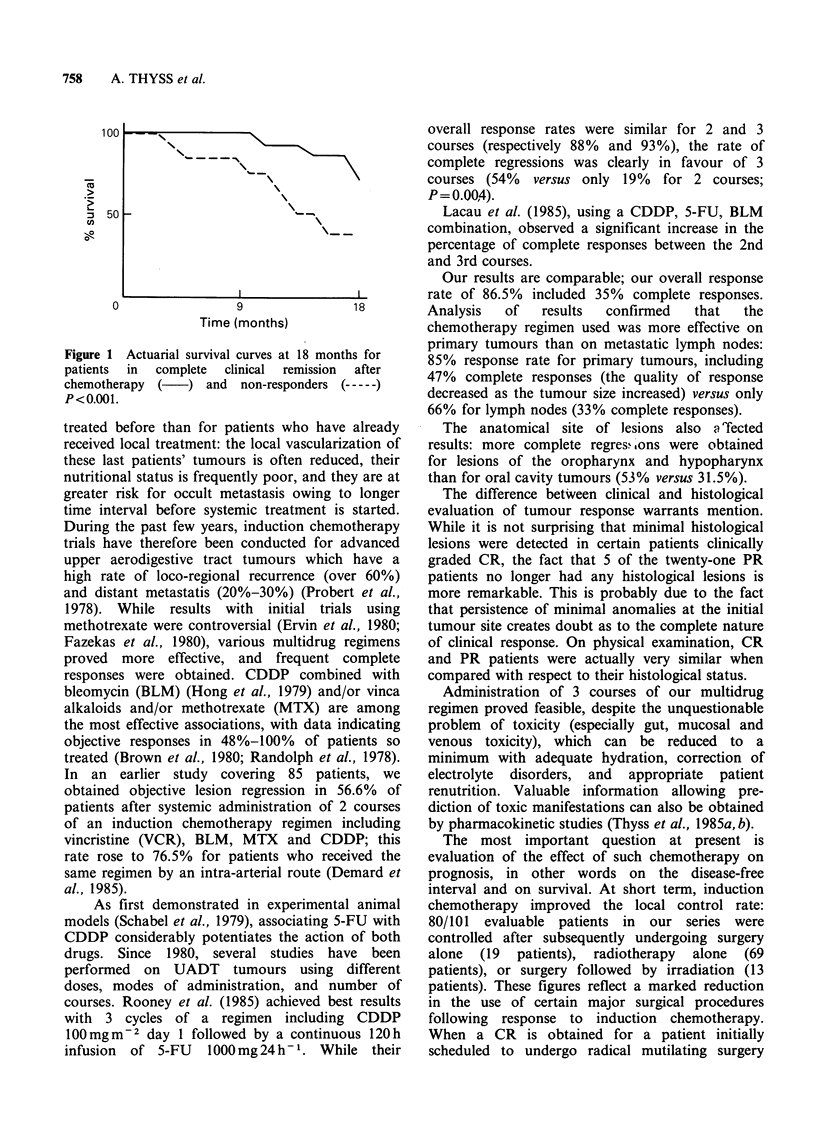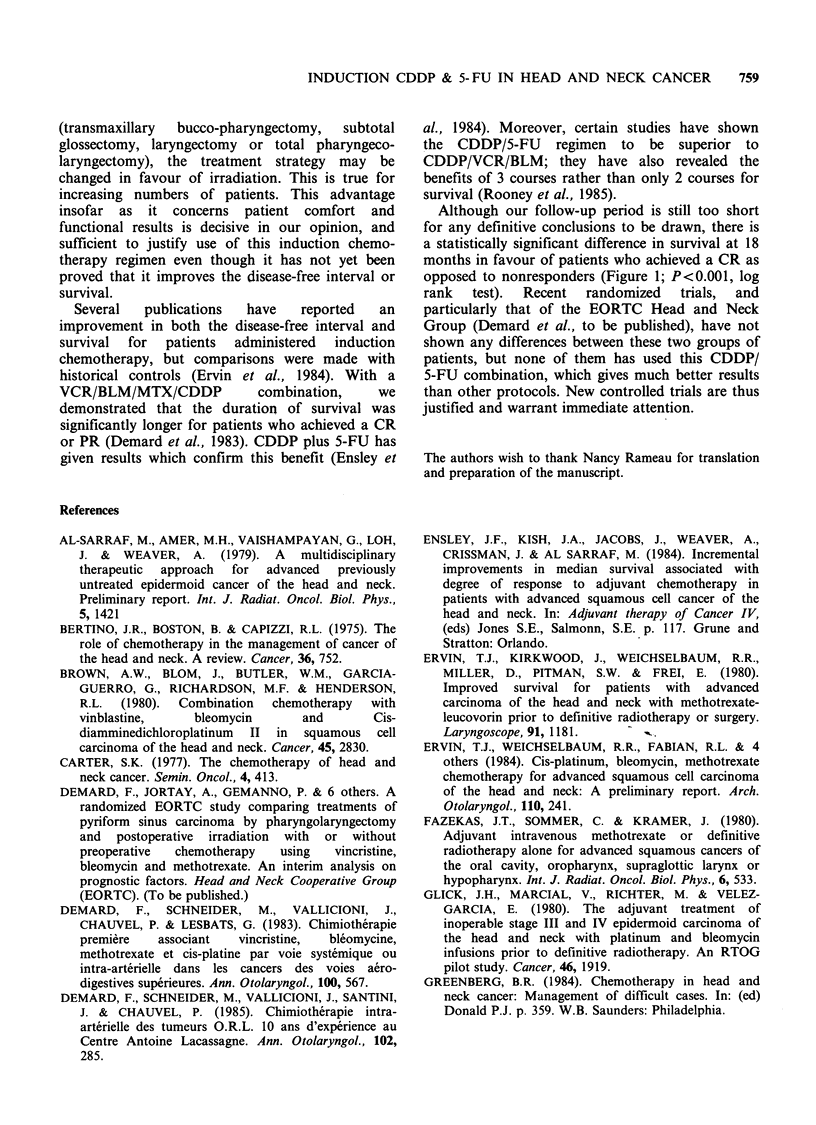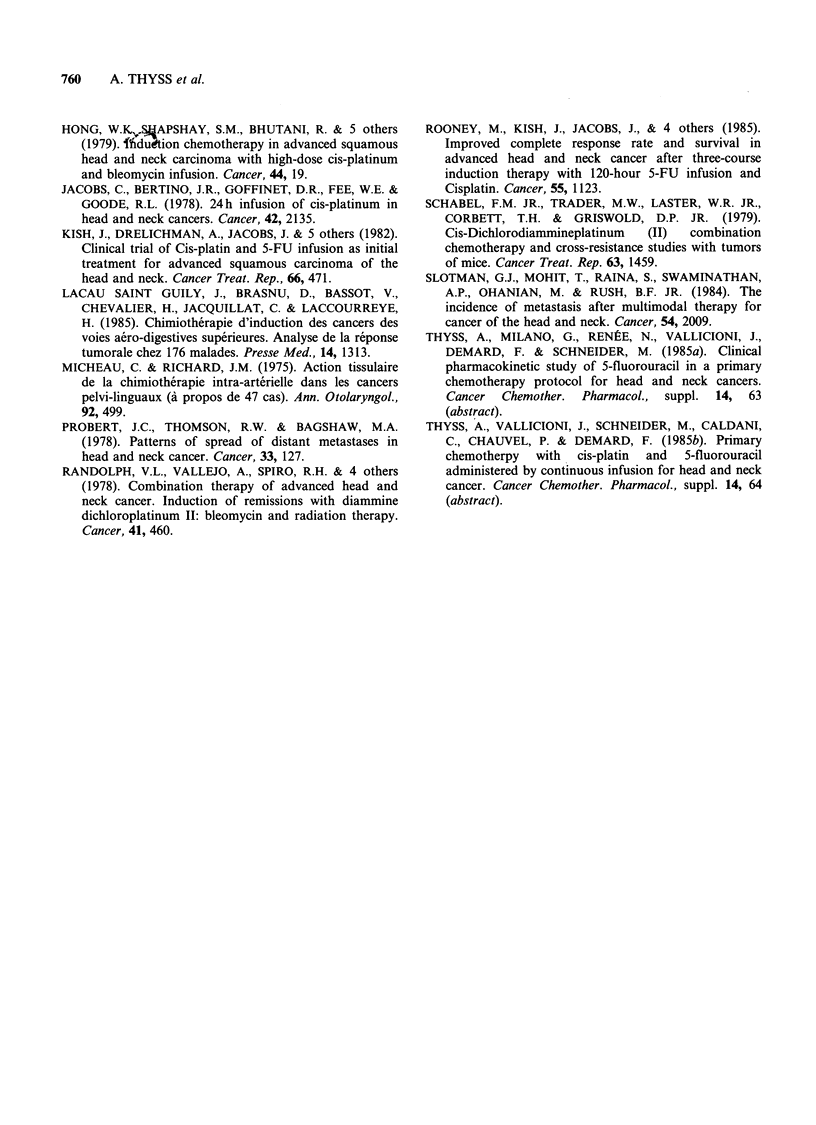# Induction chemotherapy with cis-platinum and 5-fluorouracil for squamous cell carcinoma of the head and neck.

**DOI:** 10.1038/bjc.1986.237

**Published:** 1986-11

**Authors:** A. Thyss, M. Schneider, J. Santini, C. Caldani, J. Vallicioni, P. Chauvel, F. Demard

## Abstract

One hundred and eight patients with squamous cell carcinoma of the upper aerodigestive tract (UADT) (T3, T4, NO-N3; 17% stage II, 54% stage III, 27% stage IV) were given three courses of chemotherapy before any local treatment. The regimen consisted of cis-platinum 100 mg m-2 on day 1 and 5-fluorouracil 1000 mg m-2 on days 2-6; drugs were administered by continuous infusion. The toxicity of this protocol was acceptable, as 82% of the patients were able to receive the initially scheduled drug dose. The overall response rate of 86.5% included a 35% rate of complete lesion regression. The effect of this regimen on primary tumours was especially remarkable--87.5% responses, including 47.5% complete responses. Results for lymph node metastases were not as good--66% responses, including 33% complete responses. The best results were obtained for tumours of the oropharynx and hypopharynx; oral cavity lesions were the most refractory. For those patients who were subsequently operated on, histological examination of the surgical specimen either confirmed sterilization or demonstrated the persistence of small disease foci. After local treatment, which consisted of radiotherapy alone for 69% of patients, the lesion control rate was 80%. At 18 months follow-up, the survival rate for patients who achieved a complete response with chemotherapy was significantly better than that for patients with a response of less than 50%.


*Br. J. Cancer* (1986), **54**, 755–760

### Induction chemotherapy with *cis*-platinum and 5-fluorouracil for squamous cell carcinoma of the head and neck

A. Thyss, M. Schneider, J. Santini, C. Caldani, J. Vallicioni, P. Chauvel & F. Demard

*Centre Antoine Lacassagne, 36 voie Romaine, 06054 Nice Cedex, France.*

**Summary** One hundred and eight patients with squamous cell carcinoma of the upper aerodigestive tract (UADT) (T3, T4, N0–N3; 17% stage II, 54% stage III, 27% stage IV) were given three courses of chemotherapy before any local treatment. The regimen consisted of *cis*-platinum 100 mg m$^{-2}$ on day 1 and 5-fluorouracil 1000 mg m$^{-2}$ on days 2–6; drugs were administered by continuous infusion. The toxicity of this protocol was acceptable, as 82% of the patients were able to receive the initially scheduled drug dose. The overall response rate of 86.5% included a 35% rate of complete lesion regression. The effect of this regimen on primary tumours was especially remarkable – 87.5% responses, including 47.5% complete responses. Results for lymph node metastases were not as good – 66% responses, including 33% complete responses. The best results were obtained for tumours of the oropharynx and hypopharynx; oral cavity lesions were the most refractory. For those patients who were subsequently operated on, histological examination of the surgical specimen either confirmed sterilization or demonstrated the persistence of small disease foci. After local treatment, which consisted of radiotherapy alone for 69% of patients, the lesion control rate was 80%. At 18 months follow-up, the survival rate for patients who achieved a complete response with chemotherapy was significantly better than that for patients with a response of less than 50%.

Tumours of the upper aerodigestive tract (UADT) have long been considered unresponsive to cytotoxic chemotherapy (Greenberg, 1984). For some time, methotrexate and bleomycin were essentially the only effective chemotherapeutic agents (Bertino *et al.*, 1975; Carter, 1977). These drugs were often used after local treatment (surgery and/or radiotherapy) on disease recurrence or metastasis, but results were generally disappointing. Objective response rates were significantly improved in the 1970s by the introduction of *cis*-platin, used both alone and in combination with other drugs (Jacobs *et al.*, 1978). The highest response rates were produced by induction chemotherapy given before any local treatment (Glick *et al.*, 1980).

Following the work of Al-Sarraf *et al.* (1979) and Kish *et al.* (1982), we have treated UADT tumours with an induction combination of *cis*-platinum (CDDP) and 5-fluorouracil (5-FU) since 1983. Our promising preliminary results (Thyss *et al.*, 1985) have now been confirmed by this more complete investigation of 108 cases.

### Materials and methods

### *Induction chemotherapy regimen*

Treatment consisted in continuous infusion of CDDP 100 mg m$^{-2}$ on day 1 followed by 5-FU 1000 mg m$^{-2}$ from day 2 to day 6. The protocol called for three courses per patient. The interval between cycles was 10 days for the first 63 patients (D1 = D17 = D33); the interval for subsequent patients was lengthened to 15 days (D1 = D22 = D43).

### *Criteria for admissibility*

All patients had histologically confirmed squamous cell tumours of the UADT; none had been treated previously or had metastatic disease, except metastatic cervical lymph nodes. Patients were in good general condition (WHO grade <2) and had normal renal function (creatinine <15 mg l$^{-1}$). There was no upper age limit, but drug doses were reduced 10% for every 5 years over 70 years of age.

### *Evaluation of response*

Before treatment, lesions were biopsied during endoscopy under general anaesthesia for all patients. Response was assessed by multiple biopsies obtained under identical conditions 10 days after the last chemotherapy course. The 4 patients with tumours of the sinus or face were evaluated by computed tomography. Response was defined using the products of two perpendicular lesion diameters: complete response (CR) corresponded to disappearance of all clinically visible or palpable lesions; partial response (PR) was defined as tumour regression over 50%; no response (NR)

Correspondence: A. Thyss.

Received 13 February 1986; and in revised form, 4 July 1986.

© The Macmillan Press Ltd., 1983

corresponded to tumour regression of 50% or less, stabilization, or progressive disease. The response of palpable lymph nodes was evaluated with the same criteria by physical examination and ultrasonography.

After completion of the induction chemotherapy program, all 103 patients for whom response was evaluable were given local-regional treatment: 70 patients were treated by radiotherapy alone (65–70 Gy), 33 patients underwent uni- or bilateral surgical resection completed by irradiation of the tumour bed and nodal regions (55 Gy). After loco-regional treatment, patients were examined a third time to determine the extent of control obtained by the complete therapy program. For patients who underwent surgery after chemotherapy, response was evaluated by histological examination of the surgical specimen.

*Patients*

A total of 108 patients (94 men, 14 women) were entered in the study; mean age was 61 years (range 36–82). Table I lists patient data by TNM criteria. Most patients were T3–T4 ($n=84$, 78%). The distribution between N0 (54 patients) and N2, 2, 3 (54 patients) was identical. The distribution by UICC criteria was as follows: 18 stage II (17%); 61 stage III (56%); 29 stage IV (27%). No patient had distant metastasis. The sites of primary tumours were as follows: 20 oral cavity (18%), 50 oropharynx (40%), 30 larynx or hypopharynx (28%), 4 facial sinuses (4%), 4 rhinopharynx (4%).

*Protocol feasibility*

The treatment protocol proved feasible because the doses actually administered were greater than or equal to 90% of the planned dose for 88 patients (82%). Moreover, 90 patients (83.5%) received all three chemotherapy courses, and the interval between cycles was as protocol in most cases. The interval between cycles 1 and 2 was 10 to 15 days for 81 patients (76%). For the 90 patients who received all three courses, the interval between cycles 2 and 3 had to be prolonged for more than 15 days for only 26 patients (28.5%).

**Table I** Induction chemotherapy with *cis*-platinum and 5-fluorouracil.

| *Patient distribution by tumour size and node involvement* | | | | | |
|---|---|---|---|---|---|
| | *T1* | *T2* | *T3* | *T4* | *Total* |
| N0 | – | 18 | 31 | 5 | 54 |
| N1 | – | 9 | 21 | 1 | 31 |
| N2 | – | 1 | 8 | 1 | 10 |
| N3 | 3 | 3 | 6 | 1 | 13 |
| *Patient distribution by UICC stage* | | | | | |
| Stage | II | III | IV | | |
| No. patients | 18 | 61 | 29 | | |
| Percentage | 17 | 56.5 | 27 | | |

### Results

*Overall results*

Response was evaluable for only 103 of the 108 patients; 4 patients died after the first course and 1 refused to be evaluated. Taking all lesions into account (primary tumour plus any involved nodes), an objective response was obtained in 89/103 patients (86.5%): there were 36 CR (35%), 53 PR (51.5%) and only 14 NR (13.5%).

*Results for primary tumours*

As shown by Table II, an objective response was obtained for the primary tumour in 87.5% of cases (47.5% CR); only 12.5% of patients were classed NR.

Analysis of response as a function of tumour size revealed that a CR was achieved for all 3 T1 patients (100%), for 21/31 T2 patients (68%), and for 23/62 T3 patients (37%). Although the size of the tumour at initial presentation appears to affect achievement of a CR, 2 of the seven T4 patients achieved a tumoral CR.

*Results by anatomic site*

Table III reveals that the percentage of CR was particularly high for patients with lesions of the oropharynx (53%) or hypopharynx (48%). Oral cavity tumours were more refractory to this type of chemotherapy (only 31.5% CR).

*Results for metastatic lymph nodes* (Table IV)

As is often the case, the quality of clinical response was not as good for metastatic lymph nodes as for

**Table II** Results on the primary tumour for 103 evaluable patients.

| | *Tumour size* | | | | | |
|---|---|---|---|---|---|---|
| | *T1* | *T2* | *T3* | *T4* | *Total* | % |
| Complete response | 3/3 | 21/31 | 23/62 | 2/7 | 49/103 | 47.5 |
| Partial response | – | 9/31 | 30/62 | 2/7 | 41/103 | 40 |
| No response | – | 1/31 | 9/62 | 3/7 | 13/103 | 12.5 |

**Table III** Results by anatomic site.

| | *Oral cavity* | *Oropharynx* | *Larynx-hypopharynx* |
|---|---|---|---|
| Complete response | 6 (31.5) | 24 (53) | 15 (48) |
| Partial response (>50%) | 9 (47.5) | 17 (38) | 12 (39) |
| No response | 4 (21) | 4 (9) | 4 (13) |

( )=%

**Table IV** Results on lymph node metastases.

| | *N1* | *N2* | *N3* | % |
|---|---|---|---|---|
| Complete response | 11/24 | 3/9 | 1/12 | 33 |
| Partial response (>50%) | 5/24 | 5/9 | 5/12 | 33 |
| No response | 8/24 | 1/9 | 6/12 | 33 |

primary tumours. Even so, the overall response rate of 66% (including 33% CR) is particularly high. A CR was obtained for 11 of the 24 N1 patients *versus* only 1 of the 12 N3 patients.

*Local control after completion of the treatment*

After induction chemotherapy, 19 patients were treated locally by surgery alone, 69 by radiotherapy alone, and 13 by surgery followed by radiotherapy. In all, 101 patients received the complete planned treatment: 80 were considered controlled, 13 still had progressive disease, and 8 died during the treatment program (4 during chemotherapy, 4 postoperatively).

*Evaluation of the histologic response*

The Micheau classification (Micheau & Richard, 1975) was used to evaluate tumour regression for the 31 patients who underwent surgery immediately after induction chemotherapy. For the 6 patients who had been classed CR, histologic examination of the surgical specimen confirmed CR status (IIIc) in 1 case, revealed intra-epithelial lesions in 2 cases, sterilization of the greater part of the specimen in 2 cases, and nearly complete sterilization (IIb) in the last 2 cases.

The histology report for the 21 patients who had been clinically classed PR indicated complete sterilization (IIIc) in 5 cases, sterilization of a major portion of the specimen (IIIb) in 4 cases, considerable sterilization (IIb) in 5 cases, and persistence of active lesions in 7 cases. All 4 of the NR patients who underwent surgery still had progressive disease.

*Toxicity*

Most of the patients experienced the nausea and vomiting commonly observed with treatments including *cis*-platinum. Venous damage (including pigmentation) attributable to 5-FU was also common, and was only partially prevented by keeping the infused limb in the dark. Electrolyte disorders such as hypokaliemia and hypomagnesemia were common; systematic monitoring is therefore warranted for detection and correction. Disturbances in appetite, including severe anorexia, were frequent during chemotherapy.

Haematological toxicity was evaluable for 307 cycles using WHO criteria: there were 262 grade 0 cycles (85.5%), 29 grade I or II (9.5%), and 16 grade III or IV (5%). Evaluation of gut toxicity (mucositis and/or diarrhoea) with WHO criteria gave the following results: 245 grade 0 cycles (80%), 50 grade I or II (16%), and 12 grade III or IV (4%). Most gut toxicity consisted of oral mucositis. The frequency of mucositis was reduced when the 10 day interval between cycles was increased to 15 days.

Other toxic manifestations included a transient rise in plasma creatinine, which regressed after several days for 2 patients, and balanitis for 2 other patients. Eight patients had particularly severe asthenia (6 of them were aged >70 years). Four deaths were attributable to the chemotherapy regimen, but all patients (aged 67, 70, 71 and 74 years) had significant medical complications: decompensated alcoholic cirrhosis (1 case), myocardial infarction 5 years earlier (1 case), severe arteriosclerosis of the lower limbs requiring amputation (2 cases).

Finally, in contrast to certain recent reports (Slotman *et al.*, 1984) of an increased incidence of distant metastasis in patients treated by induction chemotherapy during the 12 months following treatment, we did not observe any case of visceral metastasis after a median follow-up period of 1 year.

### Discussion

Induction chemotherapy has several theoretical advantages: Sterilization of cells that might be disseminated by surgery, reduction of the size of large tumours allowing easier local treatment, *in vivo* evaluation of tumour chemosensitivity. In addition, the rate of response to chemotherapeutic agents is higher for patients who have never been

**Figure 1** Actuarial survival curves at 18 months for patients in complete clinical remission after chemotherapy (——) and non-responders (-----) $P<0.001$.

treated before than for patients who have already received local treatment: the local vascularization of these last patients' tumours is often reduced, their nutritional status is frequently poor, and they are at greater risk for occult metastasis owing to longer time interval before systemic treatment is started. During the past few years, induction chemotherapy trials have therefore been conducted for advanced upper aerodigestive tract tumours which have a high rate of loco-regional recurrence (over 60%) and distant metastatis (20%–30%) (Probert *et al.*, 1978). While results with initial trials using methotrexate were controversial (Ervin *et al.*, 1980; Fazekas *et al.*, 1980), various multidrug regimens proved more effective, and frequent complete responses were obtained. CDDP combined with bleomycin (BLM) (Hong *et al.*, 1979) and/or vinca alkaloids and/or methotrexate (MTX) are among the most effective associations, with data indicating objective responses in 48%–100% of patients so treated (Brown *et al.*, 1980; Randolph *et al.*, 1978). In an earlier study covering 85 patients, we obtained objective lesion regression in 56.6% of patients after systemic administration of 2 courses of an induction chemotherapy regimen including vincristine (VCR), BLM, MTX and CDDP; this rate rose to 76.5% for patients who received the same regimen by an intra-arterial route (Demard *et al.*, 1985).

As first demonstrated in experimental animal models (Schabel *et al.*, 1979), associating 5-FU with CDDP considerably potentiates the action of both drugs. Since 1980, several studies have been performed on UADT tumours using different doses, modes of administration, and number of courses. Rooney *et al.* (1985) achieved best results with 3 cycles of a regimen including CDDP $100\,mg\,m^{-2}$ day 1 followed by a continuous 120 h infusion of 5-FU $1000\,mg\,24\,h^{-1}$. While their overall response rates were similar for 2 and 3 courses (respectively 88% and 93%), the rate of complete regressions was clearly in favour of 3 courses (54% *versus* only 19% for 2 courses; $P=0.004$).

Lacau *et al.* (1985), using a CDDP, 5-FU, BLM combination, observed a significant increase in the percentage of complete responses between the 2nd and 3rd courses.

Our results are comparable; our overall response rate of 86.5% included 35% complete responses. Analysis of results confirmed that the chemotherapy regimen used was more effective on primary tumours than on metastatic lymph nodes: 85% response rate for primary tumours, including 47% complete responses (the quality of response decreased as the tumour size increased) *versus* only 66% for lymph nodes (33% complete responses).

The anatomical site of lesions also affected results: more complete regressions were obtained for lesions of the oropharynx and hypopharynx than for oral cavity tumours (53% *versus* 31.5%).

The difference between clinical and histological evaluation of tumour response warrants mention. While it is not surprising that minimal histological lesions were detected in certain patients clinically graded CR, the fact that 5 of the twenty-one PR patients no longer had any histological lesions is more remarkable. This is probably due to the fact that persistence of minimal anomalies at the initial tumour site creates doubt as to the complete nature of clinical response. On physical examination, CR and PR patients were actually very similar when compared with respect to their histological status.

Administration of 3 courses of our multidrug regimen proved feasible, despite the unquestionable problem of toxicity (especially gut, mucosal and venous toxicity), which can be reduced to a minimum with adequate hydration, correction of electrolyte disorders, and appropriate patient renutrition. Valuable information allowing prediction of toxic manifestations can also be obtained by pharmacokinetic studies (Thyss *et al.*, 1985*a*, *b*).

The most important question at present is evaluation of the effect of such chemotherapy on prognosis, in other words on the disease-free interval and on survival. At short term, induction chemotherapy improved the local control rate: 80/101 evaluable patients in our series were controlled after subsequently undergoing surgery alone (19 patients), radiotherapy alone (69 patients), or surgery followed by irradiation (13 patients). These figures reflect a marked reduction in the use of certain major surgical procedures following response to induction chemotherapy. When a CR is obtained for a patient initially scheduled to undergo radical mutilating surgery

(transmaxillary bucco-pharyngectomy, subtotal glossectomy, laryngectomy or total pharyngeco-laryngectomy), the treatment strategy may be changed in favour of irradiation. This is true for increasing numbers of patients. This advantage insofar as it concerns patient comfort and functional results is decisive in our opinion, and sufficient to justify use of this induction chemotherapy regimen even though it has not yet been proved that it improves the disease-free interval or survival.

Several publications have reported an improvement in both the disease-free interval and survival for patients administered induction chemotherapy, but comparisons were made with historical controls (Ervin *et al.*, 1984). With a VCR/BLM/MTX/CDDP combination, we demonstrated that the duration of survival was significantly longer for patients who achieved a CR or PR (Demard *et al.*, 1983). CDDP plus 5-FU has given results which confirm this benefit (Ensley *et al.*, 1984). Moreover, certain studies have shown the CDDP/5-FU regimen to be superior to CDDP/VCR/BLM; they have also revealed the benefits of 3 courses rather than only 2 courses for survival (Rooney *et al.*, 1985).

Although our follow-up period is still too short for any definitive conclusions to be drawn, there is a statistically significant difference in survival at 18 months in favour of patients who achieved a CR as opposed to nonresponders (Figure 1; $P<0.001$, log rank test). Recent randomized trials, and particularly that of the EORTC Head and Neck Group (Demard *et al.*, to be published), have not shown any differences between these two groups of patients, but none of them has used this CDDP/5-FU combination, which gives much better results than other protocols. New controlled trials are thus justified and warrant immediate attention.

The authors wish to thank Nancy Rameau for translation and preparation of the manuscript.

### References

AL-SARRAF, M., AMER, M.H., VAISHAMPAYAN, G., LOH, J. & WEAVER, A. (1979). A multidisciplinary therapeutic approach for advanced previously untreated epidermoid cancer of the head and neck. Preliminary report. *Int. J. Radiat. Oncol. Biol. Phys.*, **5**, 1421

BERTINO, J.R., BOSTON, B. & CAPIZZI, R.L. (1975). The role of chemotherapy in the management of cancer of the head and neck. A review. *Cancer*, **36**, 752.

BROWN, A.W., BLOM, J., BUTLER, W.M., GARCIA-GUERRO, G., RICHARDSON, M.F. & HENDERSON, R.L. (1980). Combination chemotherapy with vinblastine, bleomycin and Cis-diamminedichloroplatinum II in squamous cell carcinoma of the head and neck. *Cancer*, **45**, 2830.

CARTER, S.K. (1977). The chemotherapy of head and neck cancer. *Semin. Oncol.*, **4**, 413.

DEMARD, F., JORTAY, A., GEMANNO, P. & 6 others. A randomized EORTC study comparing treatments of pyriform sinus carcinoma by pharyngolaryngectomy and postoperative irradiation with or without preoperative chemotherapy using vincristine, bleomycin and methotrexate. An interim analysis on prognostic factors. *Head and Neck Cooperative Group* (EORTC). (To be published.)

DEMARD, F., SCHNEIDER, M., VALLICIONI, J., CHAUVEL, P. & LESBATS, G. (1983). Chimiothérapie première associant vincristine, bléomycine, methotrexate et cis-platine par voie systémique ou intra-artérielle dans les cancers des voies aéro-digestives supérieures. *Ann. Otolaryngol.*, **100**, 567.

DEMARD, F., SCHNEIDER, M., VALLICIONI, J., SANTINI, J. & CHAUVEL, P. (1985). Chimiothérapie intra-artérielle des tumeurs O.R.L. 10 ans d'expérience au Centre Antoine Lacassagne. *Ann. Otolaryngol.*, **102**, 285.

ENSLEY, J.F., KISH, J.A., JACOBS, J., WEAVER, A., CRISSMAN, J. & AL SARRAF, M. (1984). Incremental improvements in median survival associated with degree of response to adjuvant chemotherapy in patients with advanced squamous cell cancer of the head and neck. In: *Adjuvant therapy of Cancer IV*, (eds) Jones S.E., Salmonn, S.E. p. 117. Grune and Stratton: Orlando.

ERVIN, T.J., KIRKWOOD, J., WEICHSELBAUM, R.R., MILLER, D., PITMAN, S.W. & FREI, E. (1980). Improved survival for patients with advanced carcinoma of the head and neck with methotrexate-leucovorin prior to definitive radiotherapy or surgery. *Laryngoscope*, **91**, 1181.

ERVIN, T.J., WEICHSELBAUM, R.R., FABIAN, R.L. & 4 others (1984). Cis-platinum, bleomycin, methotrexate chemotherapy for advanced squamous cell carcinoma of the head and neck: A preliminary report. *Arch. Otolaryngol.*, **110**, 241.

FAZEKAS, J.T., SOMMER, C. & KRAMER, J. (1980). Adjuvant intravenous methotrexate or definitive radiotherapy alone for advanced squamous cancers of the oral cavity, oropharynx, supraglottic larynx or hypopharynx. *Int. J. Radiat. Oncol. Biol. Phys.*, **6**, 533.

GLICK, J.H., MARCIAL, V., RICHTER, M. & VELEZ-GARCIA, E. (1980). The adjuvant treatment of inoperable stage III and IV epidermoid carcinoma of the head and neck with platinum and bleomycin infusions prior to definitive radiotherapy. An RTOG pilot study. *Cancer*, **46**, 1919.

GREENBERG, B.R. (1984). Chemotherapy in head and neck cancer: Management of difficult cases. In: (ed) Donald P.J. p. 359. W.B. Saunders: Philadelphia.

HONG, W.K., SHAPSHAY, S.M., BHUTANI, R. & 5 others (1979). Induction chemotherapy in advanced squamous head and neck carcinoma with high-dose cis-platinum and bleomycin infusion. *Cancer*, **44**, 19.

JACOBS, C., BERTINO, J.R., GOFFINET, D.R., FEE, W.E. & GOODE, R.L. (1978). 24 h infusion of cis-platinum in head and neck cancers. *Cancer*, **42**, 2135.

KISH, J., DRELICHMAN, A., JACOBS, J. & 5 others (1982). Clinical trial of Cis-platin and 5-FU infusion as initial treatment for advanced squamous carcinoma of the head and neck. *Cancer Treat. Rep.*, **66**, 471.

LACAU SAINT GUILY, J., BRASNU, D., BASSOT, V., CHEVALIER, H., JACQUILLAT, C. & LACCOURREYE, H. (1985). Chimiothérapie d'induction des cancers des voies aéro-digestives supérieures. Analyse de la réponse tumorale chez 176 malades. *Presse Med.*, **14**, 1313.

MICHEAU, C. & RICHARD, J.M. (1975). Action tissulaire de la chimiothérapie intra-artérielle dans les cancers pelvi-linguaux (à propos de 47 cas). *Ann. Otolaryngol.*, **92**, 499.

PROBERT, J.C., THOMSON, R.W. & BAGSHAW, M.A. (1978). Patterns of spread of distant metastases in head and neck cancer. *Cancer*, **33**, 127.

RANDOLPH, V.L., VALLEJO, A., SPIRO, R.H. & 4 others (1978). Combination therapy of advanced head and neck cancer. Induction of remissions with diammine dichloroplatinum II: bleomycin and radiation therapy. *Cancer*, **41**, 460.

ROONEY, M., KISH, J., JACOBS, J., & 4 others (1985). Improved complete response rate and survival in advanced head and neck cancer after three-course induction therapy with 120-hour 5-FU infusion and Cisplatin. *Cancer*, **55**, 1123.

SCHABEL, F.M. JR., TRADER, M.W., LASTER, W.R. JR., CORBETT, T.H. & GRISWOLD, D.P. JR. (1979). Cis-Dichlorodiammineplatinum (II) combination chemotherapy and cross-resistance studies with tumors of mice. *Cancer Treat. Rep.* **63**, 1459.

SLOTMAN, G.J., MOHIT, T., RAINA, S., SWAMINATHAN, A.P., OHANIAN, M. & RUSH, B.F. JR. (1984). The incidence of metastasis after multimodal therapy for cancer of the head and neck. *Cancer*, **54**, 2009.

THYSS, A., MILANO, G., RENÉE, N., VALLICIONI, J., DEMARD, F. & SCHNEIDER, M. (1985*a*). Clinical pharmacokinetic study of 5-fluorouracil in a primary chemotherapy protocol for head and neck cancers. *Cancer Chemother. Pharmacol.*, suppl. **14**, 63 (*abstract*).

THYSS, A., VALLICIONI, J., SCHNEIDER, M., CALDANI, C., CHAUVEL, P. & DEMARD, F. (1985*b*). Primary chemotherpy with cis-platin and 5-fluorouracil administered by continuous infusion for head and neck cancer. *Cancer Chemother. Pharmacol.*, suppl. **14**, 64 (*abstract*).